# Pangenome analysis indicates evolutionary origins and genetic diversity: emphasis on the role of nodulation in symbiotic *Bradyrhizobium*

**DOI:** 10.3389/fpls.2025.1539151

**Published:** 2025-04-02

**Authors:** Leonardo Araujo Terra, Milena Serenato Klepa, Marco Antonio Nogueira, Mariangela Hungria

**Affiliations:** ^1^ CNPq, Brasília, Brazil; ^2^ Soil Biotechnology Laboratory, Embrapa Soja, Londrina, Paraná, Brazil

**Keywords:** *Bradyrhizobium*, pangenome, biogeography, Nod factors, Fix/Nif, type III secretion system, nodulation, environmental adaptation

## Abstract

The *Bradyrhizobium* genus is widely known for encompassing many species capable of forming nodules and establishing the biological nitrogen fixation process with several legumes, significantly contributing to agriculture and environmental sustainability. Despite its importance, questions about the evolution, pangenome, and symbiotic genes of *Bradyrhizobium* are still poorly understood. In this study, we analyzed the pangenome of a set of *Bradyrhizobium* symbiotic species using the Roary and GET_HOMOLOGUES tools in strains originated from the Northern and Southern Hemispheres. We also investigated the presence and correlation of the *fix*, *nif*, *nod*, Type III secretion system (T3SS) and their effector proteins, and T4SS genes, trying to find differences between clades, hosts, and biogeographic origin. Pangenome analysis of *Bradyrhizobium* species from the Northern and Southern Hemispheres provided valuable insights into their diversity, biogeography, origin, and co-evolution with their legume host plants. The genus possesses a relatively small core genome compared to the expanded accessory genome, a key feature that facilitates genetic exchange and acquisition of new genes, allowing adaptation to a variety of environments. Notably, the presence or absence of T3SS effector proteins varied significantly according to the geographic location, suggesting specific environmental adaptations, as well as a direct relationship with nodulation genes. Comparative analysis indicated that symbiotic *Bradyrhizobium* species originated in the Northern Hemisphere and present a greater diversity of orthologous groups than those from the Southern Hemisphere. These results contribute to our understanding of the evolutionary history of these symbiotic bacteria.

## 1 Introduction

Legumes represent a family of plants that are widely distributed around the world and have coevolved with nitrogen-fixing symbiotic bacteria for millions of years (Doyle, 2011; Sprent et al., 2017). The wide variety of adaptations resulting from this coevolution between plants and bacteria has allowed the occupation of diverse ecosystems, from arid regions to tropical forests (Werner et al., 2014). The geographic distribution of legumes and their close relationships with symbiotic-nodulating bacteria, such as those of the *Bradyrhizobium* genus, reveal patterns related to evolutionary history and biogeography, adapting to different environmental conditions and playing important roles in ecosystems and agriculture (Parker, 2012; Sprent et al., 2017).

Species belonging to the genus *Bradyrhizobium* are cosmopolitan bacteria that occupy diverse ecosystems and habitats around the world. The biogeography of these bacteria plays a fundamental role in adapting to a variety of global environments (Hungria et al., 2015; Sprent et al., 2017; Bouznif et al., 2019), contributing to the maintenance of ecosystems, sustainability, and agricultural productivity (Hungria et al., 2015).

Within the genus *Bradyrhizobium*, several species depend on nodulation (*nod*) genes that encode Nod factors, signaling molecules essential for host plant recognition and nodule formation in legume roots (Ormeño-Orrillo and Martínez-Romero, 2019). This genus is also an important target for studies on *nif* (nitrogen fixation) and *fix* genes that encode proteins essential for biological nitrogen fixation (BNF). The *nif* genes are responsible for the synthesis and functioning of nitrogenase, an enzyme complex that converts molecular nitrogen (N_2_) into ammonia (NH_3_^+^), while the *fix* genes act in the regulation and maintenance of nitrogenase activity (Menna and Hungria, 2011). Furthermore, the *Bradyrhizobium* genus is also studied in relation to nodulation genes (*nod*), Nod Factors, the Type III Secretion System (T3SS) and its effector proteins (T3E), which play fundamental roles in nodule formation (Giraud et al., 2007; Teulet et al., 2022).

With the establishment of symbioses, biological nitrogen fixation takes place, and it is recognized as the second most important process for plant growth after photosynthesis (Hungria and Nogueira, 2023). In general, phylogenetic and genomic analyses of rhizobial strains have provided valuable information about their evolution and geographic distribution (Stępkowski et al., 2007; Avontuur et al., 2019).

Advances in DNA sequencing technologies have enabled the generation of data from sequenced bacterial genomes, enabling pangenome analyses, which represent a valuable tool for exploring genetic diversity within bacterial species (McInerney et al., 2017; Brockhurst et al., 2019). The concept of pangenome encompasses all genes identified in a set of strains or representants of a given taxonomic group. It considers genes shared by all strains or species, known as the core genome and represented by genes predicted to encode common and vital functions, as well as genes that occur exclusively in a few organisms, representing the accessory genome and conferring the ability to adapt to different lifestyles. This approach reveals the intrinsic genomic diversity of a bacterial species or genus and the ability to adapt to different ecological environments (Tettelin et al., 2008; Brockhurst et al., 2019).

Some studies have explored the pangenomes of strains of the same species, for example, *Escherichia coli* (Rasko et al., 2008) and *Pseudomonas aeruginosa* (Mosquera-Rendón et al., 2016), of species within a genus, e.g., *Burkholderia* (Bach et al., 2022), *Rhizobium* (González et al., 2019), or even at the order level, for example, *Rhizobiales* (Rosselli et al., 2021), and the results obtained have advanced the understanding of these microorganisms. An analysis of the *Burkholderia* pangenome showed the presence of genes acquired via HGT from other microorganisms, contributing to their ability to survive in the rhizosphere (Bach et al., 2022). Similarly, a pangenome analysis of *Bradyrhizobium* revealed genetic traits associated with nitrogen fixation, highlighting the role of genetic diversity in shaping symbiotic efficiency (Zhong et al., 2024). Furthermore, the modular nature of symbiotic genes in *Bradyrhizobium* allows for evolutionary stability and flexibility, with horizontal gene transfer (HGT) playing a key role in the maintenance and reorganization of symbiotic traits (Weisberg et al., 2022).

In this study, we investigated the genetic diversity of the *nif*/*fix*, nod and T3SS genes and their effector proteins, as well as the biogeographic distribution of *nod*-dependent of *Bradyrhizobium* species isolated from legume nodules around the world. Through pangenome analysis, we sought to understand how these genes are organized and how their variations are associated with the different environments of the Northern and Southern Hemispheres. Our results provide new insights into the genetic diversity and evolution of these bacteria, highlighting the differences associated with the biogeography of the species in each hemisphere.

## 2 Materials and methods

### 2.1 Selection of symbiotic *Bradyrhizobium* species

A total of 54 genomes from different *Bradyrhizobium* species were retrieved from the GenBank database on 02/04/2023, based on well-documented geographic identification. Genome selection followed the following criteria: (1) presence of *Bradyrhizobium* strains isolated from root nodules of wild or cultivated legumes; (2) presence of *nif*/*fix* genes; and (3) presence of nod genes. In addition, genomes from phylogenetically distinct strains, unusually numerous single genes, and reduced number of genes in the accessory genome were excluded from the analysis. Based on these criteria, a total of 44 symbiotic nitrogen-fixing *Bradyrhizobium* genomes dependent on nod factors were selected. Of these, 11 were completely sequenced, while 33 were draft genomes (**Supplementary Table S1**).

### 2.2 Pangenome

#### 2.2.1 Pangenome construction

To construct the pangenome, we used two programs: Roary, with option “-i 90” (v3.13.0) (Page et al., 2015), and GET_HOMOLOGUES, with option “-M -t 0 -i90” (v15052022) (Contreras-Moreira and Vinuesa, 2013). The GFF3 and gbk files of annotated genomes from Prokka (v1.14.6) (Seemann, 2014) were used as inputs for Roary and GET_HOMOLOGUES, respectively. Genome completeness and contamination were assessed via CheckM v.1.2.1 (Parks et al., 2015) and assembly quality parameters were analyzed via QUAST v.5.2.0 (Gurevich et al., 2013).

#### 2.2.2 Core genome phylogeny

The phylogenetic tree was built on the basis of the core clusters identified via the CoreCruncher tool (Harris et al., 2021). Protein sequences with 90% identity, sizes of 80%, and frequencies of 100% were aligned via the –MAFFT (v7.490) program (Katoh and Standley, 2013) and concatenated. Multiple sequence alignment was used to construct phylogenetic trees via the LG+G+I+F model in MEGA 11 software (v11.0.13) (Tamura et al., 2021), with a bootstrap value of 100.

For phylogeographic analysis, alignments obtained from the core genome were analyzed using the BEAST tool (v1.10.4). Inference was performed with the Blosum62 substitution model with the Strict Clock molecular clock model and Symmetric CTMC (Continuous-Time Markov Chain) phylogeographic model. The population structure was modeled using a coalescent model, while geographic states were defined as Northern Hemisphere (N) and Southern Hemisphere (S). Bayesian inference was conducted with a Markov Chain Monte Carlo (MCMC) of 10 million iterations and Effective Sample Size (ESS) values greater than 200 (**Supplementary Figure S1**).

### 2.3 Prediction of Fix/Nif, Nod, T3SS, Type III effectors and T4SS clusters

The protein identification data were obtained from the GenBank database via the National Center for Biotechnology Information (NCBI) website in April 2023. The sequences of proteins involved in biological nitrogen fixation (Fix/Nif) (**Supplementary Table S2**), nodulation (Nod) (**Supplementary Table S3**), and Type III effector proteins were searched in the NCBI database, extracted from the reference genomes of *B. diazoefficiens* USDA110, *B. elkanii* USDA61, and *Sinorhizobium fredii* HH103 and used to identify homologous proteins via the BLASTP Local tool (Altschul et al., 1990). We set the parameters of 50% coverage and 50% identity. The MacsyFinder (v2.1) (Abby et al., 2014) tool with default settings and hmm TXSS profiles were used to annotate the Type III and Type IV secretion systems (**Supplementary Table S4**).

### 2.4 Enrichment analysis of gene clusters

The Scoary v. 1.6.16 (Brynildsrud et al., 2016) tool with the options “–collapse - and 100” was used to perform genome-wide association study (GWAS) analysis of the gene content present in the *B. jicamae*, *B. elkanii*, and *B. japonicum* clades regarding biogeography. The output files with 100% specificity were used as criteria to filter the proteins.

Accessory genome sequences were extracted via the Seqkit (v2.3.0) tool (Shen et al., 2016). The functional annotation of pangenomes was performed with the EggNOG-mapper (Huerta-Cepas et al., 2017). COG and KEGG annotations were derived from the EggNOG mapper results. We considered a function enriched if the *p* value and *q* value were less than 0.05, which controlled the expected proportion of false positives at 0.05.

Insertion sequences present in the genome were assessed via genome annotations and ISESCAN 1.7.2.3 (Xie and Tang, 2017).

### 2.5 Statistical analyses

Plots of the core and accessory genomes were generated via the R package Pagoo (Ferrés and Iraola, 2021). Histograms were generated via the native R package. UpSet graphs were generated via the UpSetR package (Conway et al., 2017). Gene presence/absence heatmaps were generated via the R package pheatmap. The map was generated via the matplotlib library, and the interactive map was generated via the folium library via the Python language.

## 3 Results

### 3.1 Phylogeny of the core genome and genomic distribution of the *fix*, *nif*, *nod*, T3SS, and T3E genes

**Figure 1:**
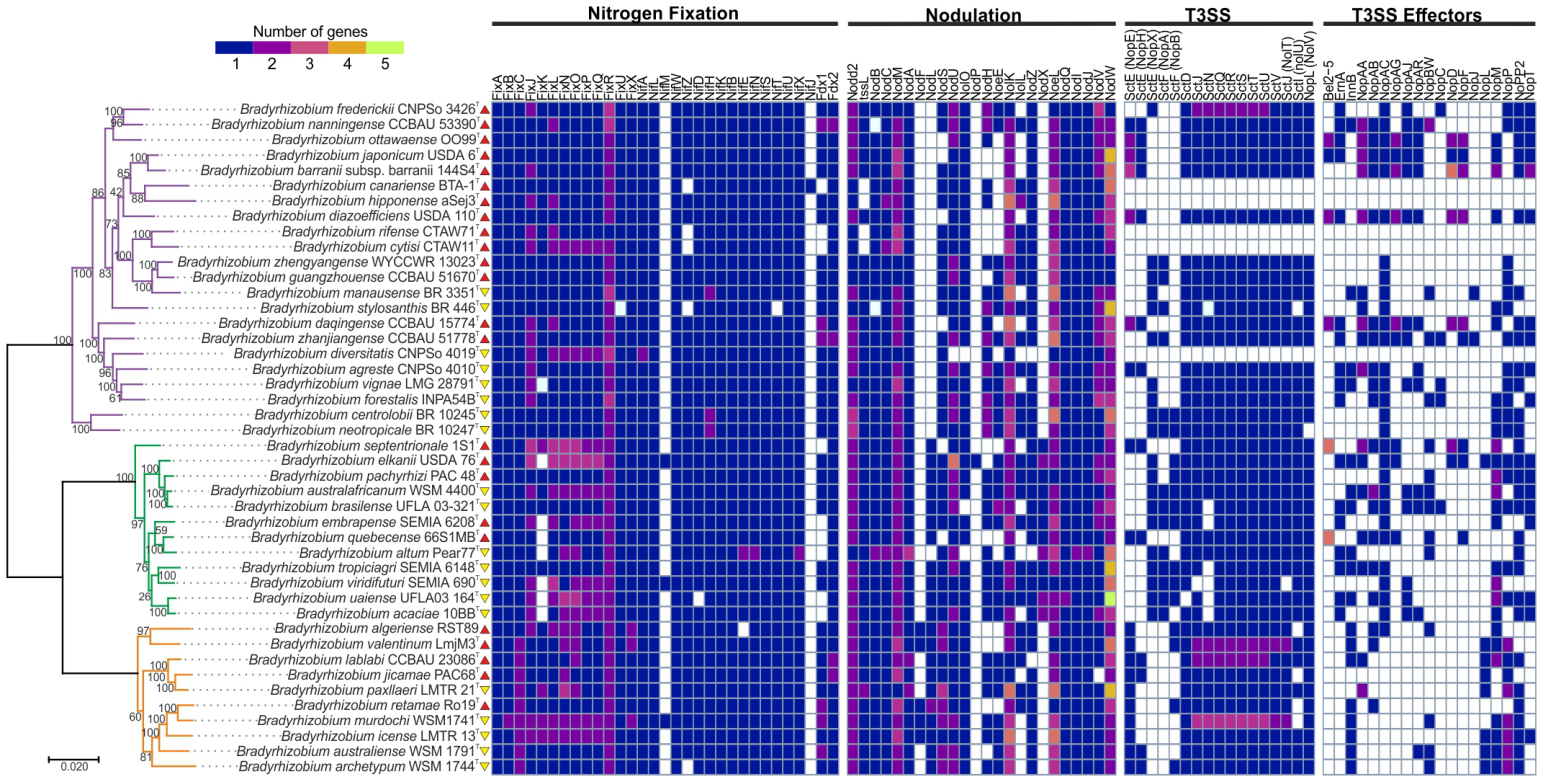
Representation of the absence and presence of genes involved in the metabolism of biological nitrogen fixation, nodulation, the T3SS, and candidate genes for T3SS and T3E effectors in symbiotic *Bradyrhizobium*. The phylogenetic tree is classified according to the clades: *B*. *japonicum* (purple), *B*. *elkanii* (green), and *B*. *jicamae* (orange). Red upward arrows indicate bacteria isolated in the Northern Hemisphere, whereas yellow downward arrows indicate bacteria isolated in the Southern Hemisphere. The colors in the heatmap represent the number of proteins found in each strain, following a variation scale. The scale bar represents the number of amino acid substitutions per site.

The phylogenetic tree constructed from 163 concatenated core proteins separated the *Bradyrhizobium* species under study into two superclades, one with a unique clade, *B. japonicum*, and the second with two clades, *B. elkanii* and *B. jicamae* (**Figure 1**).

We found conserved genes associated with the *Bradyrhizobium* genome: *fix*, *nif*, *nod*, T3SS, and their effector proteins. It is worth to mention that from the 44 genomes studied here, 34 belong to at least 10 different symbiovars (Martinez-Romero et al., 2024) (**Supplementary Table S1**). A detailed analysis of these genes revealed that 11% of the *Bradyrhizobium* strains did not exhibit T3SS genes in their genomic repertoire (**Figure 1**).

The presence and absence of *fix*, *nif*, *nod* and T3SS genes, as well as their effector proteins and T4SS were correlated with clade, geographic distribution and/or host plant (**Supplementary Figures S2–S16**). In genes related to biological nitrogen fixation, a low occurrence of the FixK protein, positive regulator that controls nitrogenase activity, was observed in the *B. elkanii* clade, whereas its presence was confirmed in all strains of the *B. jicamae* and *B. japonicum* clades, except for the strain *B. vignae* LMG 28791 of the *B. japonicum* clade. The FixR protein, oxidoreductase activity, was abundant in all clades with multiple copies, except for *B. rifense* with only one copy. Among the Nod proteins, NodF protein, acyl carrier, was detected only in some members of the *B. jicamae* clade,. while the NodX protein, O-acetyltransferase, was found only in the *B. elkanii* clade. The NodL protein, O-acetyltransferase, is absent in the *B. japonicum* clade. Regarding T3SS proteins and their effector proteins, NopA protein and the T3E proteins Bel2-5, ErnA, NopA, NopAG, NopAJ, NopAC, NopC, NopD, NopF, and NopJ were not identified in the *B. jicamae* clade, whereas InnB and NopL were widely found in this clade.

Regarding the geographic distribution and hosts, *Bradyrhizobium* isolated from *Chamaecytisus prolifer*, *Cytisus villous* and *Lupinius angustifolius*, plants located in North Africa, do not present T3SS genes and their effectors. The only exception is *B. diversitatis*, isolated from soybean (*Glycine max*) in Australia, which also does not present these genes. Interestingly, all these strains belong to the *B. japonicum* clade. The absence of T3SS and its effector proteins indicates that some *Bradyrhizobium* species can establish symbiosis exclusively through Nod Factors. Furthermore, the presence of T3SS can activate immunity caused by effects on certain host plants, which would result in the infection of the symbiosis by the plant.

With respect to the genes related to their hosts and the geographic distribution of the T3SS proteins and their effector proteins, Bel2-5 and NopD proteins were exclusive to the soybean plant (*Glycine max*), and the NopAG and NopF proteins were predominant in this host plant. The NopE, NopH, NopAA, and NopT proteins were predominant in the Northern Hemisphere. In the Southern Hemisphere, the NopX, NopA, InnB, and NopP proteins were predominant. The NopP2 protein is commonly distributed in all clades and hemispheres.

The transcriptional regulator gene *ttsL*, which is important for T3SS activation in the nodulation process, was identified in all the genomes containing T3SS genes. The absence of both *ttsL* and T3SS genes may indicate simultaneous horizontal transfer of these elements. The exception was *B. diversitatis* CNPSo 4019, which lacks the T3SS but carries the *ttsL* gene, which may indicate another biological function for this gene (**Figure 1**).

In particular, among the proteins associated with the T3SS structure, strains that possess the NopX protein do not present NopE or NopH, and vice versa. The presence of NopX is strongly correlated with that of NopA, suggesting a possible biological interaction. Furthermore, NopX is absent in the *B. jicamae* clade. On the other hand, NopE and NopH proteins are predominant in the *B. japonicum* clade and present a strong correlation with the SctN protein (**Figure 1**).

Interestingly, our results indicate that the presence of NopE, NopH and NopX proteins is related to host specificity, indicating that different legume hosts have a preference for these proteins. Furthermore, the NopX protein occurs exclusively in species from Brazil, South Africa, and Colombia and in some species from Central America, the USA and China. This protein is absent in species from Canada, North Africa, and Oceania. The NopE and NopH proteins are predominant in North America, Asia and Oceania (See Interactive Map, **Supplementary File 3**).

### 3.2 Pangenome of symbiotic *Bradyrhizobium* species

#### 3.2.1 Selection of organisms

The set of 44 *Bradyrhizobium* genomes consisted of incomplete genome assemblies with contigs and scaffolds and completely sequenced genomes. All other genomes had completeness higher than 97.17% and contamination below 3.11%, ensuring that the genomes were of adequate quality for pangenome analysis (**Supplementary Table S1**).

#### 3.2.2 Pangenome analysis

**Figure 2:**
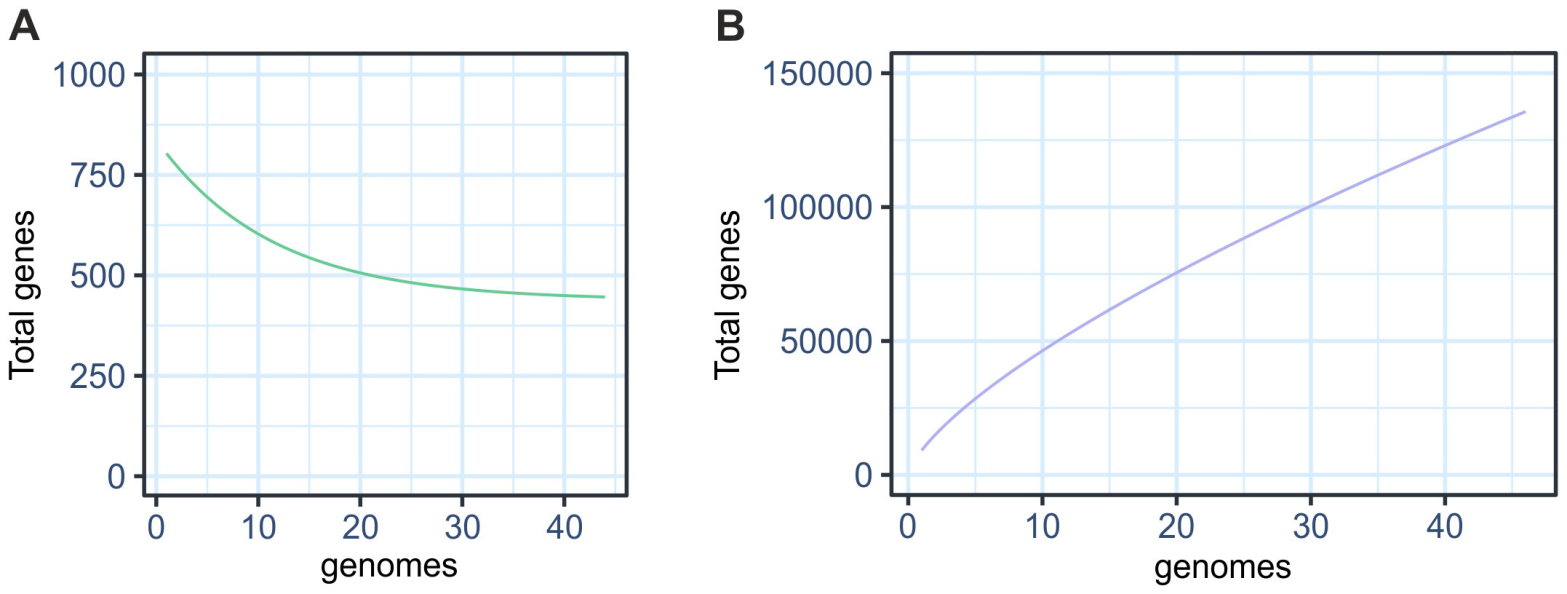
Pangenome characteristics of 44 species of symbiotic *Bradyrhizobium*. **(A)** Core genome curve **(B)** Accessory genome curve.

Pangenome analysis identifies whether a given taxon has an open or closed genome. The central genes represent the core genome, which are the genes shared by all strains analyzed. This curve shows a decline as new genes are added, indicating a small set of genes strictly conserved in this taxon (**Figure 2A**). On the other hand, the accessory genome includes variable genes present in some strains and absent in others. The continuous growth of this curve indicates that new accessory genes continue to be added to the pangenome as more genomes are incorporated (**Figure 2B**). Our results indicate that the total number of gene sets increased without reaching a plateau, characterizing an open pangenome.

Analysis of the Roary pangenome revealed a total of 127,128 gene families, close to the 133,100 gene families detected with the GET_HOMOLOGUES. **Table 1** presents the number of genes in the pangenome categorized into core, soft-core, shell, and cloud categories via sequence alignment with 90% identity. The results revealed similarities in the absolute distribution values between the core, soft-core, and shell categories.

**Table 1:** Distribution of pangenomes classified by Roary and GET_HOMOLOGUES.

Genes	GET_HOMOLOGUES	Roary
Core* (99 – 100%) = 44 genomes	386	438
Soft core (95 – 99%) 42 <= strains <= 44 genomes	577 (386 + 191)	524 (438 + 86)
Shell genomes (15 – 95%) 7 <= strains < 41 genomes	12,825	13,357
Cloud (0 – 15%) 6 genomes	119,698	113,247
Pangenome (total)	133,100	127,128

*Core genome: Genes present in all species.

Soft core genome: Genes found in most species, usually used for incomplete genomes.

Shell genome: Genes shared by most species, but not all. Cloud genome: Rare genes, present in few species.

#### 3.2.3 Northern and Southern Hemisphere pangenomes

##### 3.2.3.1 Genetic interaction of the accessory genome

Genome separation between the Northern (latitude ≥ 0°) and Southern (latitude < 0°) Hemispheres was performed to investigate the impact of biogeography on the evolution and genetic diversity of *Bradyrhizobium* species dependent on nod factors. Although the exact origin of *Bradyrhizobium* is still uncertain, comparing genomes between hemispheres may provide insights into its diversification and dispersal.

In the Northern Hemisphere, 9 complete and 15 incomplete genomes were analyzed, with an average completeness of 99.69%. In the Southern Hemisphere, 2 complete and 18 incomplete genomes were identified, with an average of 99.51%. Although the proportion of incomplete genomes is higher in the Southern Hemisphere, the high average completeness in both groups indicates that the analyses of orthologous groups remain reliable and representative.

The geographic distribution of the genomes analyzed spans several continents in different hemispheres. In North Africa, 5 genomes were analyzed, while in South Africa, 4 genomes were analyzed. In North America, 7 genomes were distributed between Canada and the United States. In Central America, 2 genomes were analyzed, distributed in Honduras and Costa Rica.

In South America, a total of 12 genomes were analyzed, with the majority of records originating from Brazil and some from Peru and Colombia. In Australia, 5 genomes were analyzed, reinforcing the representativeness of Oceania. In Asia, the number of genomes identified was 7, covering different regions of China and Japan. Finally, 2 genomes were counted, with samples from Spain.

**Figure 3:**
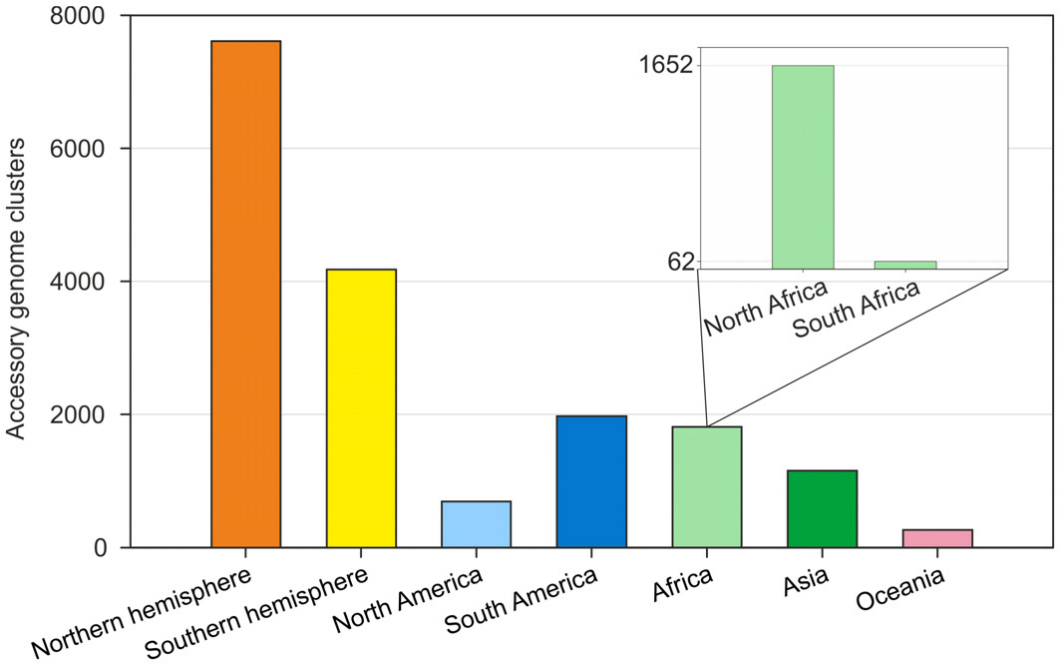
Global distribution of orthologous clusters of the *Bradyrhizobium* accessory genome by geographic region and between hemispheres. The main graph highlights the hemisphere groupings and specific regions. The secondary graph highlights the African-specific accessory genome clusters, highlighting the specificity of accessory genomes between North Africa and South Africa.

In the Northern Hemisphere, despite having only four more species than in the Southern Hemisphere did, the accessory genomes presented 1.8 times more orthologous groups, totaling 4,128 clusters between North America, North Africa, and Asia, suggesting high genetic interaction between these species. Notably, species from North Africa share 1,652 orthologous groups, indicating greater genetic interaction than species from South Africa (**Figure 3**).

**Figure 4:**
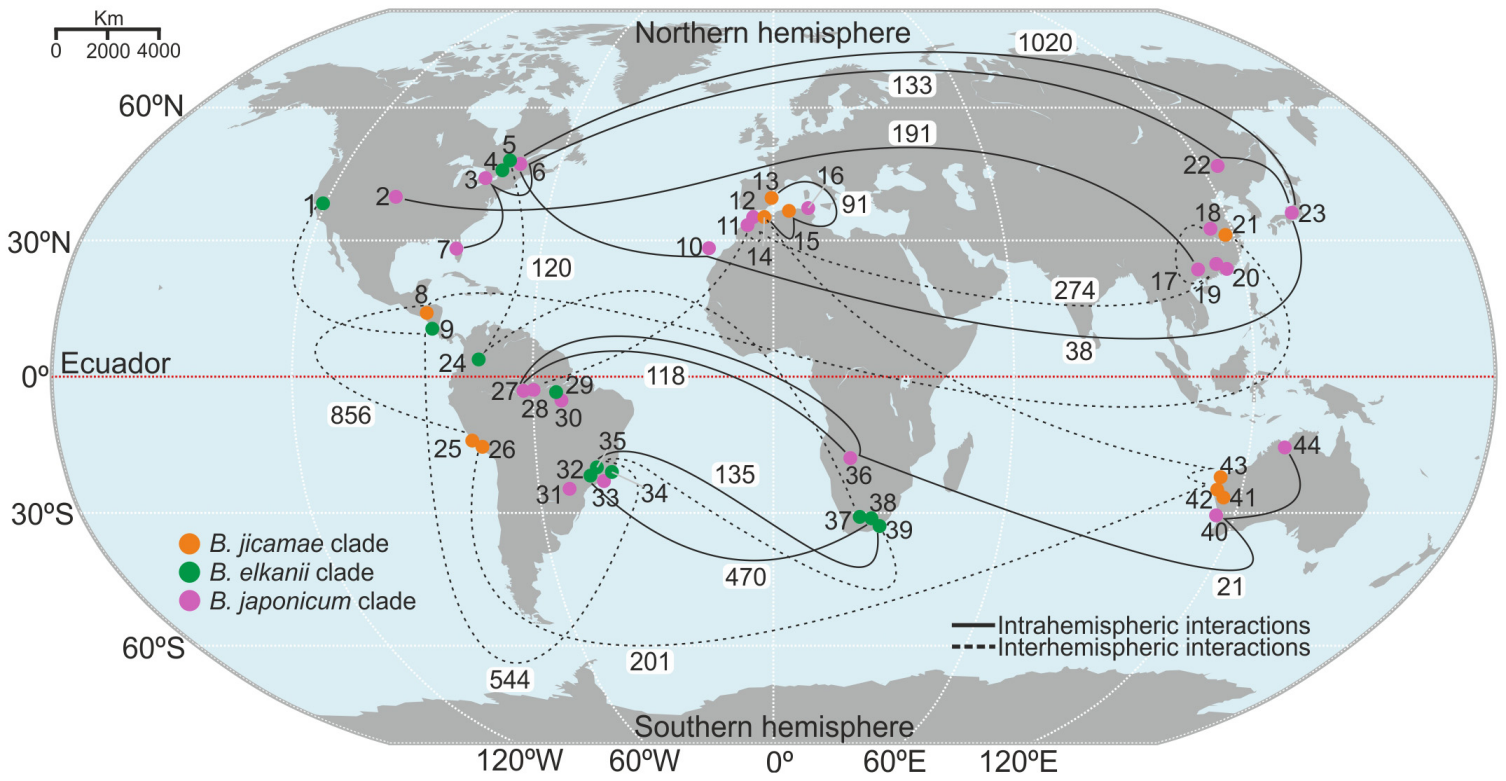
Global distribution and genetic interaction among *Bradyrhizobium* species, according to the accessory genome. White boxes indicate the sharing of accessory genome gene clusters. Each species is identified by a number: 1. *Bradyrhizobium elkanii;* 2. *B. frederickii;* 3. *B. ottawaense;* 4. *B. septentrionale;* 5. *B. quebecense;* 6. *B. baranii;* 7. *B. diazoefficiens;* 8. *B. jicamae;* 9. *B. pachyrhizi;* 10. *B. canariense;* 11. *B. rifense;* 12. *B. cytisi;* 13. *B. valentinum*; 14. *B. retamae;* 15. *B. algeriense*; 16. *B*. *hipponense*; 17. *B*. *nanningense*; 18. *B*. *zhengyangense*; 19. *B*. *guangzhouense*; 20. *B*. *zhanjiangense*; 21. *B*. *lablabi;* 22. *B. daqingense;* 23. *B. japonicum;* 24. *B*. *embrapense;* 25. *B. paxllaeri*; 26. *B*. *icense*; 27. *B*. *forestalis*; 28. *B*. *manauense*; 29. *B*. *tropiciagri;* 30. *B*. *centrolobii;* 31. *B. neotropical*; 32. *B. uaiense;* 33. *B. brasilense;* 34. *B. stylosanthis*; 35. *B*. *viridifuturi;* 36. *B. vignae*; 37. *B. altum;* 38. *B. acaciae;* 39. *B. australafricanum;* 40. *B. diversitatis*; 41. *B*. *archetypum;* 42. *B. australiense*; 43. *B*. *murdochi;* 44. *B. agreste*.

Genetic interactions related to the accessory genome were investigated among species from regions between the Northern and Southern Hemispheres. We identified a robust genetic relationship between species from North America and Asia and between species from Spain, the Canary Islands (a Spanish archipelago in the Atlantic Ocean close to North Africa), and North Africa (**Figure 4**). In contrast, genetic interactions between species from Asia, the Canary Islands, and North America were more tenuous (**Supplementary Figure S17**). In the Southern Hemisphere, strong genetic interactions were detected between species from Brazil and South Africa. However, the interaction between Oceania and South America was moderate (**Supplementary Figure S18**).

Genetic interactions between the hemispheres that cross the equator were investigated. The *B. jicamae* clade revealed a genetic interaction pattern connecting North Africa, Oceania, and South America, which indicates a potential gene flow route in this geographical location. In the *B. elkanii* clade, it is possible to observe a gene flow path in North America, extending through Central and South America and reaching South Africa. The *B. japonicum* clade included a genetic pathway extending from Asia, North Africa, and South America. Notably, few genetic interactions were detected between species from North Africa and South Africa (**Supplementary Figure S19**).

##### 3.2.3.2 Orthologous clusters

**Figure 5:**
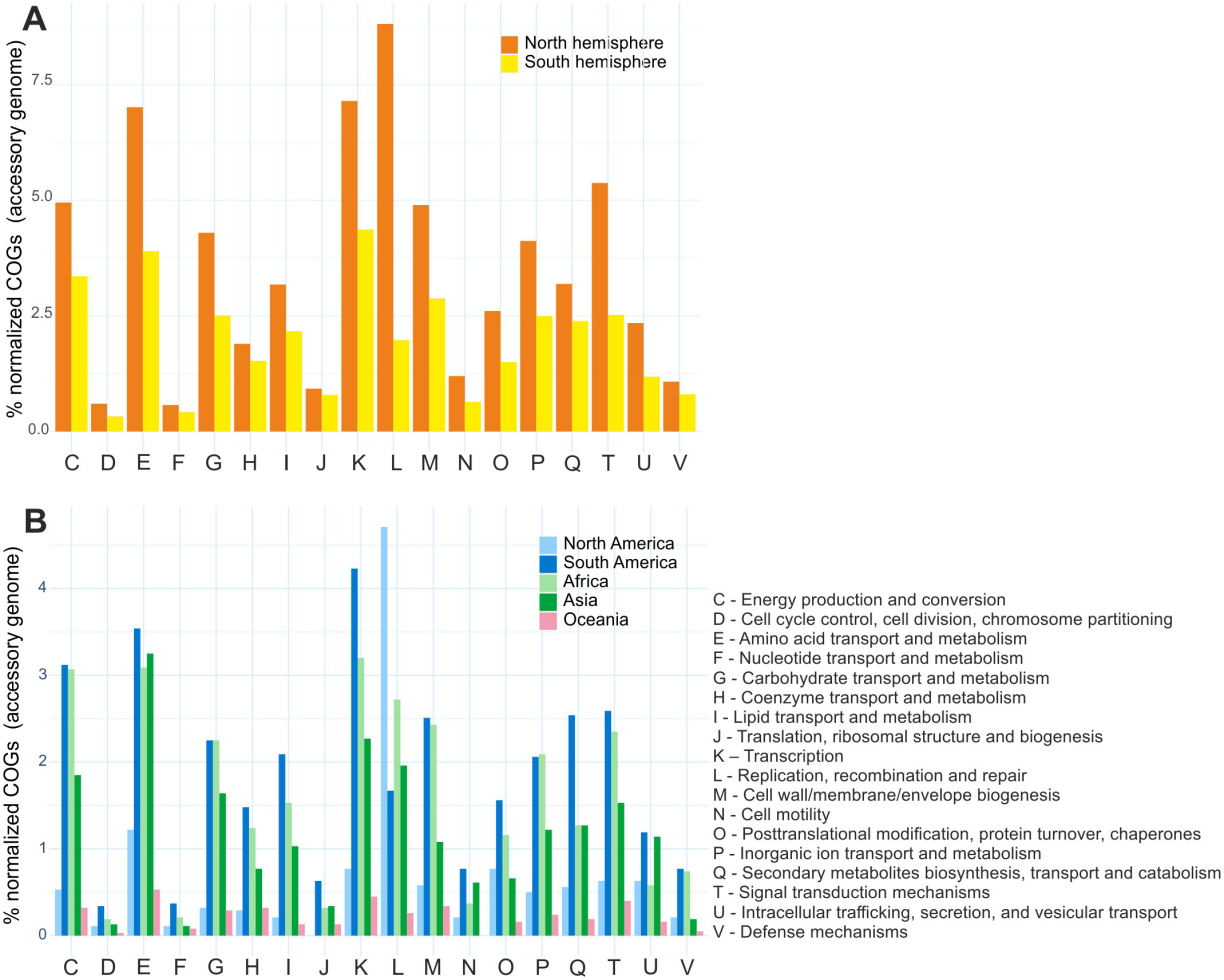
Functional distribution of accessory genes in the pangenome according to COG categories: **(A)** Northern and Southern Hemisphere groups. **(B)** Specific subgroups of species isolated in North America, South America, Africa, Asia, and Oceania.

Comparative analysis of clusters of orthologous groups (COGs) between *Bradyrhizobium* species from the Northern and Southern Hemispheres revealed different distributions of gene clusters in the accessory genome. A predominance of clusters associated with functions such as replication, recombination and repair (L), transcription (K), amino acid metabolism and transport (E), and signal translation mechanisms (T) was detected in Northern Hemisphere species. In the Southern Hemisphere, transcription (K) and metabolism and transport of amino acids (E) are highlighted, as are groups involved in energy conversion and production (C) (**Figure 5**). It is important to highlight that the species from the Northern Hemisphere presented a high quantity of transposases within the functional group L.

A detailed analysis of the clusters showed that species from North America and Oceania presented a reduced number of gene clusters. Notably, species from North America showed a high presence of replication, recombination and repair (L) clusters, suggesting an evolutionary strategy to maintain genomic plasticity. In contrast, species from South America, North Africa and Asia exhibited a greater richness of genes associated with transcription (K), amino acid metabolism and transport (E) and energy conversion and production (C) (**Figure 5**).

A detailed analysis of the insertion elements corroborated the COG category findings in replication, recombination and repair (L) (**Supplementary Figure S20**).

## 4 Discussion

In this study, specific representatives of each *Bradyrhizobium* species capable of forming nodules from different continental regions were selected, allowing us to investigate the influence of biogeography on the evolution and genetic diversity of these microorganisms. The genetic framework of these genomic representatives includes genes involved in adaptations to different water stresses, changes in pH and temperature, and interactions with their host plants (Freitas et al., 2022; Zhang et al., 2024). These characteristics impose selective pressures on these microorganisms, capturing genomic signatures that are direct results of the environmental pressure from which the organisms were isolated. Pangenome analysis revealed that species isolated in the Northern Hemisphere showed greater sharing of accessory genes compared to the Southern Hemisphere, indicating greater genetic interaction between species in this region. This difference may be associated with ecological factors such as environmental and host diversity, which may have favored greater gene exchange. The abundance of transposases and insertion elements in species from the Northern Hemisphere suggests greater genomic plasticity, which may have facilitated the adaptation of these species to different environments. Furthermore, by selecting genomes from different biogeographic environments, it was possible to understand the conservation of genes critical for local adaptation, evidencing the evolutionary pressure on the diversification of *Bradyrhizobium*.

Our pangenome study revealed a reduced core genome and a large accessory genome, evidencing broad genetic variability and the ability to adapt to various environments. Our results are in line with the studies by Weisberg et al. (2022) and Zhong et al. (2024) who showed that *Bradyrhizobium* has a small core genome and an extensive accessory genome, which characterizes an open pangenome. This genomic profile favors the acquisition of new genes via HGT, favoring adaptation to different environmental conditions. The genomic plasticity shown in these studies demonstrates the ability of *Bradyrhizobium* to colonize a wide variety of hosts.

Molecular evidence from fossils indicates that legumes emerged approximately 60 million years ago (Ma) between the Cretaceous and Cenozoic periods (Herendeen, 1992). At that time, Laurasia comprised a continuous and extensive landmass in the Northern Hemisphere. On the other hand, paleogeographic evidence indicates that during the fragmentation of Gondwana, South America remained connected to Antarctica. These facts disagree with the hypothesis of an African origin of legumes, favoring the boreotropic theory that suggests a Northern Hemisphere origin of legumes, with subsequent southward dispersal (see Figure 4 of Doyle and Luckow, 2003). This interpretation is supported by terrestrial connectivity between Africa and Eurasia during the Eocene and the subsequent union of the Americas (3-4 Ma), offering a more coherent overview of migration routes.

The analysis of the *Bradyrhizobium* accessory genome revealed a broad interaction of orthologous clusters between Asia and North America, suggesting intense genetic exchange in the Northern Hemisphere (**Figure 4**). Furthermore, our results revealed a putative gene flow route from *B. elkanii* from North America through Central America and South America that extends to southern Africa (**Figure 4**). According to the boreotropic hypothesis, legume dispersion occurs via continental masses, and these plants act as biotic vectors for rhizobia dispersal (Doyle and Luckow, 2003). Furthermore, despite the geological separation of continents before the emergence of legumes, it is possible to observe genetic interactions between *Bradyrhizobium* species from southern Africa, Oceania, and South America (**Figure 4**). According to Vinuesa and collaborators (Vinuesa et al., 2005), when studying the migration and speciation of *B. canariense* and *B. japonicum*, the most likely means for the transcontinental and hemispheric dispersal of *Bradyrhizobium* cells are by abiotic vectors, such as dust masses. Soil carrying *Bradyrhizobium*, which is transported by wind currents, has been reported in Brazil, and dust storms can reach large distances, favoring bacterial dispersal (Ferreira and Hungria, 2002). This hypothesis is reinforced by the results of our studies, which revealed genetic interactions over long distances, covering isolates from Asia, North Africa, and North America. In parallel, we observed short-range genetic interactions between *Bradyrhizobium* species from the *B. jicamae* and *B. japonicum* clades, which were specifically isolated from legumes of the Genistae tribe in the regions of North Africa, the Canary Islands, and Spain (See Interactive Map, **Supplementary File 3**).

We identified limited genetic interactions between species from North Africa and South Africa, possibly because they contain different biomes, raising significant questions about the dispersal routes of these species across landmasses over time and challenging the two hypotheses presented.

Schrire et al. (2005) carried out extensive work on the biogeography of legumes, revealing many differences between the hemispheres. In the Northern Hemisphere, the distribution of biomes is more homogeneous, favoring greater gene flow and facilitating dispersal and interbreeding between populations. In contrast, the Southern Hemisphere has a greater diversity of biomes, including tropical forests, savannas, and succulent and temperate biomes. This fragmentation of biomes promoted more specific selective pressures, reducing gene flow between populations. Furthermore, their work mentioned that the first legumes evolved in a semiarid region north of the Tethys Sea route, which separated the supercontinents Laurasia and Gondwana.

Our pangenome results for *Bradyrhizobium* indicate greater genetic sharing of the accessory genome in the Northern Hemisphere than in the Southern Hemisphere, corroborating the biogeographic patterns observed in legumes. These results indicate a possible origin of *Bradyrhizobium* in the Northern Hemisphere and close and adaptive coevolution with legumes over time (**Figure 3**, **4**, **Supplementary Figure S1**). *Bradyrhizobium* has been suggested as the ancestor of all rhizobia (Provorov and Vorob’ev, 2000; Lloret and Martínez-Romero, 2005; Hungria et al., 2015); therefore, a Northern Hemisphere origin would also indicate that bacterial symbiosis originated in this region of the world.

The geological connection between Australia and Antarctica, as part of Gondwana, allowed the migration of plant species between Australia and South America. This connection is evidenced by the presence of the Brongniartieae tribe, a monophyletic clade present on both continents (Sprent et al., 2013). This relationship may clarify the genomic interactions between members of the *B. jicamae* clade inhabiting Australia and South America.

Some studies have reported that several legume species found in South America, previously considered archaic, are native to North America (Taylor, 1990; Lavin and Luckow, 1993), indicating that legume species in South America are evolutionarily more recent. These studies align with the phylogenetic analysis (**Supplementary Figure S1**), which revealed that most ancestral nodes are associated with the Northern Hemisphere, supporting the idea that *Bradyrhizobium* species likely originated in this region before dispersing to the Southern Hemisphere.

The genomes of *Hyphomicrobiales* (=*Rhizobiales*), including *Bradyrhizobium*, are marked by an abundance of insertion elements (Minamisawa et al., 1998; Barros-Carvalho et al., 2019; Bender et al., 2022). These elements are fundamental for the movement of DNA segments, contributing to genomic plasticity and diversity. Our analysis of the pangenome revealed the widespread presence of transposases and insertion elements in the Northern Hemisphere, suggesting progress in the evolutionary process and genomic adaptation compared with those in the Southern Hemisphere. Furthermore, components of the T4SS genetic framework were more abundant in the Northern Hemisphere. The identification of these genetic elements more concentrated in the Northern Hemisphere strains supports the hypothesis that they may have greater nodulation capacity through horizontal gene transfer, a key mechanism for the adaptation and evolution of these symbiotic organisms (Sprent, 1994).

The α-rhizobial T3SS genes and *nod* genes are coregulated, and their activation is triggered by plant and bacterial flavonoids. In the presence of flavonoids, bacterial NodD activates *nod* genes, and the regulator TtsL promotes the expression of T3E proteins (Dong and Song, 2020). Studies with nodule isolates belonging to the genus *Bradyrhizobium* have indicated that 90% of the T3SS genes are conserved (Teulet et al., 2020), confirming the identification rate of 89% of these genes in the species of this study. Species lacking the T3SS are specific to the *B. japonicum* clade. Furthermore, the presence of up to three noncanonical T3SS clusters in some species suggests a functional complexity that still needs to be understood (Tampakaki, 2014), and further studies are needed to elucidate these multiple T3SS clusters. Symbiovars are groups of strains that share similar symbiotic capabilities, supported by *nodC* and *nifH* phylogenies (Rogel et al., 2011). Here, species belonging to the same symbiovar generally exhibit the same T3SS and *nod* gene repertoire (**Figure 1**; **Supplementary Table S1**), supporting Teulet et al. (2020)’s suggestion of a common evolutionary origin for these genes.

The NopA and NopB proteins form needle-shaped extracellular filaments (Teulet et al., 2020), whereas NopX, NopH, and NopE act as translocons anchored inside the cell, facilitating the entry of effector proteins into host cells. Although NopE is recognized as a translocon (Wenzel et al., 2010), the exact function of NopH still requires further study, although this protein has been found in the secretome of *B. diazoefficiens* USDA110^T^ (Staehelin and Krishnan, 2015). Teulet et al. (2022) proposed that the NopH and NopE proteins perform functions similar to those of NopX in T3E translocation. Our results indicate that the presence of NopX is related to the absence of NopH and NopE, suggesting that these two proteins may have compensatory functions in effector translocation, acting as alternatives when NopX is not present.

*Bradyrhizobium* species associated with Genisteae legumes include populations from the Canary Islands, North Africa and Spain, which exhibit different profiles regarding the type III secretion system (T3SS) and the TtsL regulator. We observed that species lacking both the T3SS and the TtsL regulator or those that simultaneously present the T3SS with the NopE and NopB proteins are associated with Genisteae legumes (see Interactive Map). In addition, these species, *B. canariense, B. hipponense, B. rifense*, and *B. cytisi*, are grouped in the symbiovar genistearum (Martinez-Romero et al., 2024). Pangenome studies with *Bradyrhizobium* confirmed that most isolates from Genisteae legume tribe lack the T3SS (Zhong et al., 2024). Interestingly, the species lacking the T3SS in this group possess the T4SS (see Interactive Map). A notable example is *B. canariense*, which lacks the T3SS and TtsL regulator but is able to nodulate several Genisteae and Loteae legumes (Vinuesa et al., 2005), suggesting an evolutionary advantage in these geographic regions. Studies involving the type IV (T4SS) and type VI (T6SS) secretion systems in *Bradyrhizobium* demonstrated the ability to induce nodulation in legumes, possibly compensating for the absence of the T3SS in the establishment of symbiosis (Banerjee et al., 2019; Tighilt et al., 2022; Wangthaisong et al., 2023).

The T3SS of *Bradyrhizobium* is encoded by a gene cluster known as *Rhizobium*-conserved (Rhc), whose evolution is characterized by several gains and losses, indicating specific adaptations (Teulet et al., 2020). The interaction between the NopA and NopX proteins suggests that a specific biological relationship was absent in the *B. jicamae* clade. These components are predominantly found in strains from the Southern Hemisphere, especially in Brazil, South Africa, and Colombia, whereas NopX is absent in strains from Canada, North Africa, and Oceania. The distribution patterns of the RhcIa and RhcIb clusters, which are associated with NopE/NopH and NopX/NopA, respectively, highlight the dynamic evolution of *Bradyrhizobium* (Teulet et al., 2020), suggesting that adaptations are influenced by symbiosis with host plants and are related to biogeography.

Variations in the composition of T3SS effector proteins may represent fundamental adaptations for bacteria to avoid plant immune responses or establish specific interactions and play a role in the transport and translocation of bacterial effector proteins into host plant cells (Staehelin and Krishnan, 2015; Miwa and Okazaki, 2017). The T3E proteins ErnA, NopAC, NopM, NopP, NopP2, and NopT are present in most of the genomes analyzed in our study. The NopM, NopP, and NopT proteins are found in other rhizobial genera (Kimbrel et al., 2013), and Teulet et al. (2020) proposed that these proteins were acquired horizontally by the ancestors of *Bradyrhizobium* lineages together with the T3SS cluster and then lost in some lineages.

In our study, NopAG and NopF proteins were predominantly found in *Bradyrhizobium* isolated from soybean (*Glycine max*), whereas the Bel2-5 and NopD proteins were exclusive to this plant. The NopAG, Bel2-5 and NopD proteins play important roles in inhibiting plant defenses, facilitating symbiosis, and influencing the processing of small plant ubiquitin proteins (Xiang et al., 2020).

The analysis of genomic relationships revealed that *B. japonicum* and *B. daqigense*, which were isolated in Asia, indicated that they have accessory genomes related to *B. diazoefficiens*, *B. barrani*, and *B. ottawaense* from North America. The presence of common T3E in all these genomes, with the exception of *B. barranii*, which lost the Bel2-5 and ErnA proteins, highlights the importance of these effectors in symbiotic interactions. These T3Es are essential to suppress plant defense responses and allow effective colonization (Teulet et al., 2022). The absence of these effectors in *B. barranii* suggests that this lineage may have adopted other strategies to survive and develop in the environment, possibly compensating for the loss with other symbiotic mechanisms or adjusting to different ecological niches.

## 5 Conclusion

Our study expands our understanding of the biogeography and evolution of symbiotic species of the genus *Bradyrhizobium*, providing evidence on their geographic origin and gene flow. Phylogeographic analysis of the core genome revealed that most ancestral lineages were associated with the Northern Hemisphere, indicating this region as the likely origin of these organisms.

In addition, pangenome data indicated a more intense gene flow between species from the Northern Hemisphere compared to the Southern Hemisphere. Our investigation also revealed a diversity of genes associated with symbiotic functions, including *fix*, *nif*, *nod*, and the T3SS and its effector proteins, demonstrating that these genes are correlated with the phylogenetic clades, hosts, and biogeographic conditions in which the species occur.

## Data Availability

The original contributions presented in the study are included in the article/**Supplementary Material**. Further inquiries can be directed to the corresponding author.

## Supplementary material

Supplementary File 1 (**Tables S1, S2, S3 and S4)** | Description of the selected genomes, including the identification and selection of *fix*/*nif* and *nod* genes related to nitrogen fixation and symbiosis, genes associated with the type III secretion system (T3SS) and their effector proteins, and the type IV secretion system (T4SS).

Supplementary File 2
**(Figures S1, S2, S3, S4, S5, S6, S7, S8, S9, S10, S11, S12, S13, S14, S15, S16, S17, S18 and S19)** | Heat map representing the distribution of *fix*/*nif*, *nod*, T3SS and their effector proteins and T4SS genes in comparison to the different hemispheres, clades (*Bradyrhizboium jicamae, B. elkanii and B. japonicum*) and their respective hosts. UpSet plots demonstrating the sharing of accessory genome gene clusters between species from the Northern and Southern Hemispheres. Analysis of the distribution of insertion elements in *Bradyrhizobium* analyzed by different geographic regions and comparisons between hemispheres.

Supplementary File 3 Instruction guide for using the interactive map.
