# Supplementary figures and images for: Pangenome analysis indicates evolutionary origins and genetic diversity: emphasis on the role of nodulation in symbiotic Bradyrhizobium
Source: Front Plant Sci. 2025 Apr 2;16:1539151. doi: 10.3389/fpls.2025.1539151 (PMC12000093; doi:10.3389/fpls.2025.1539151)

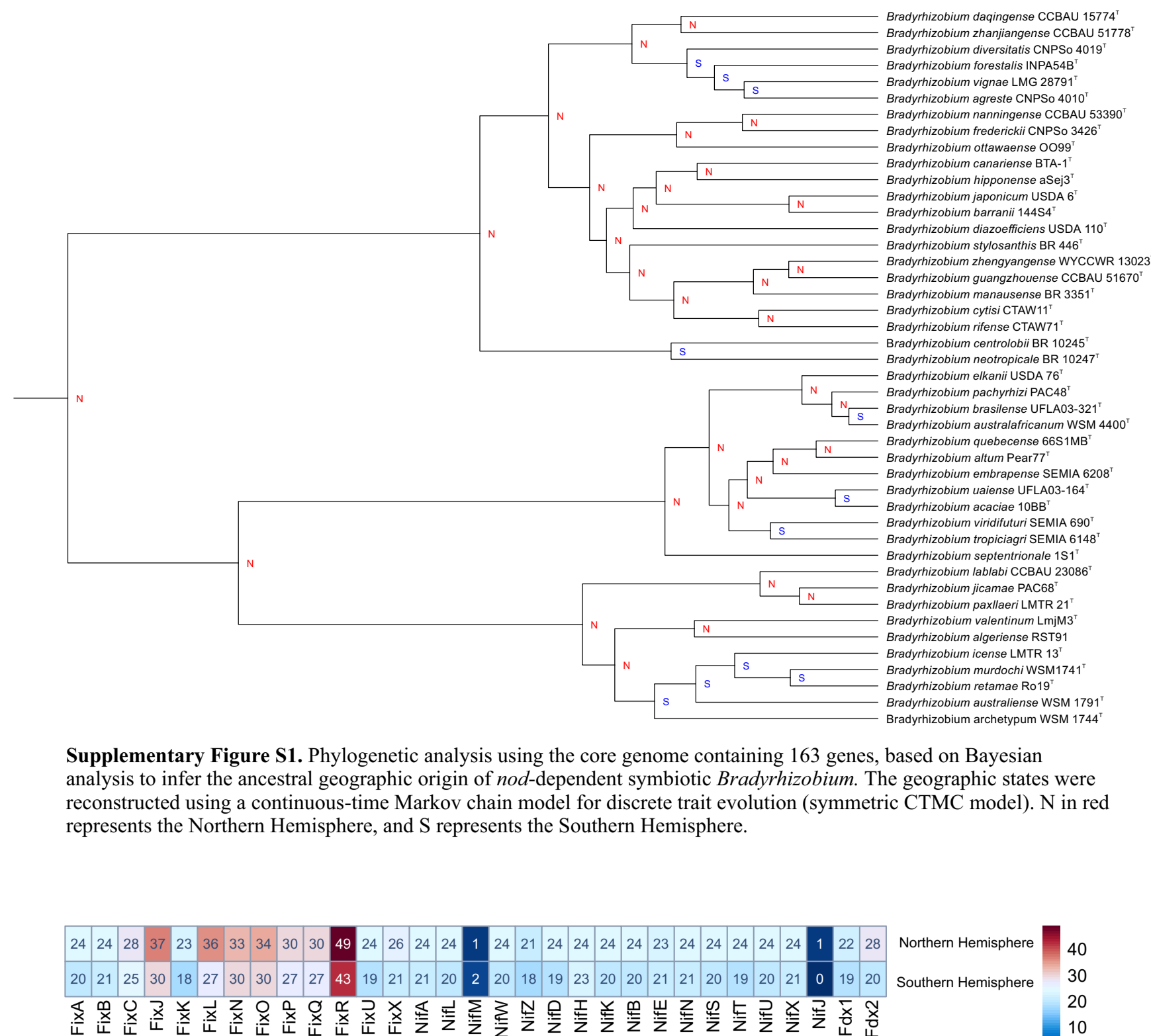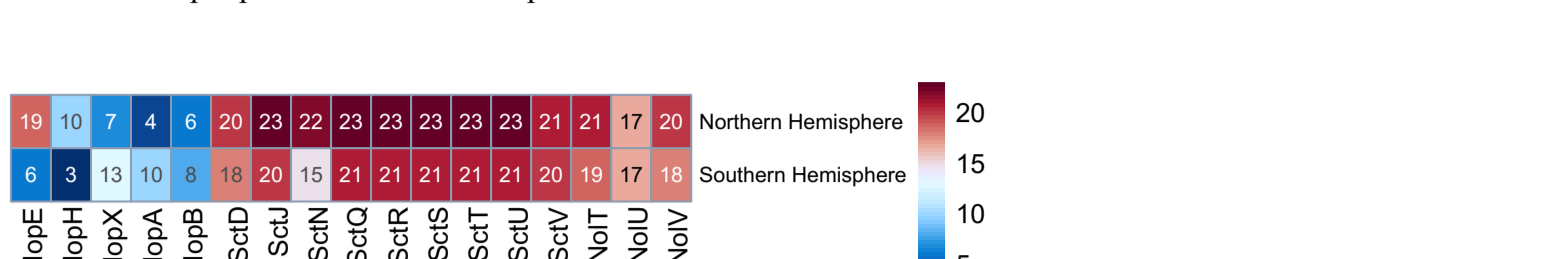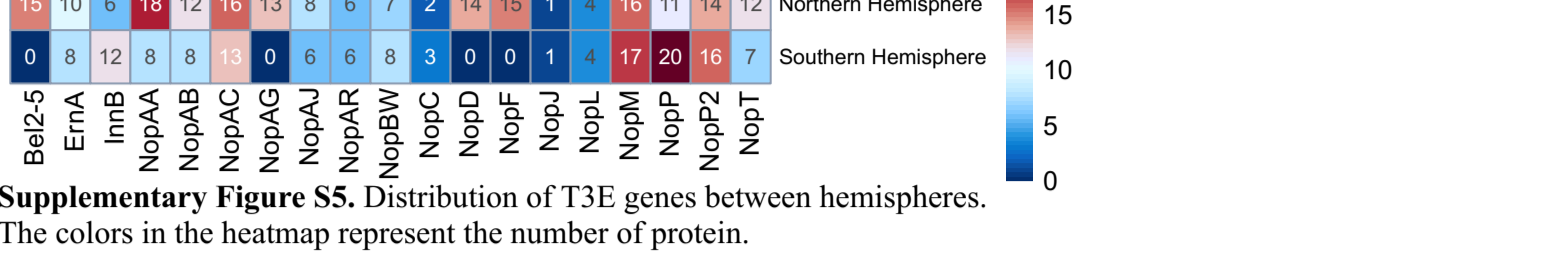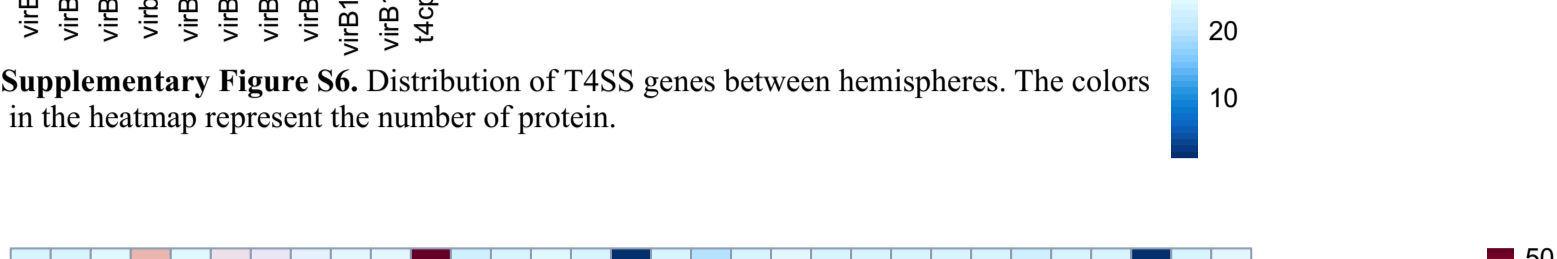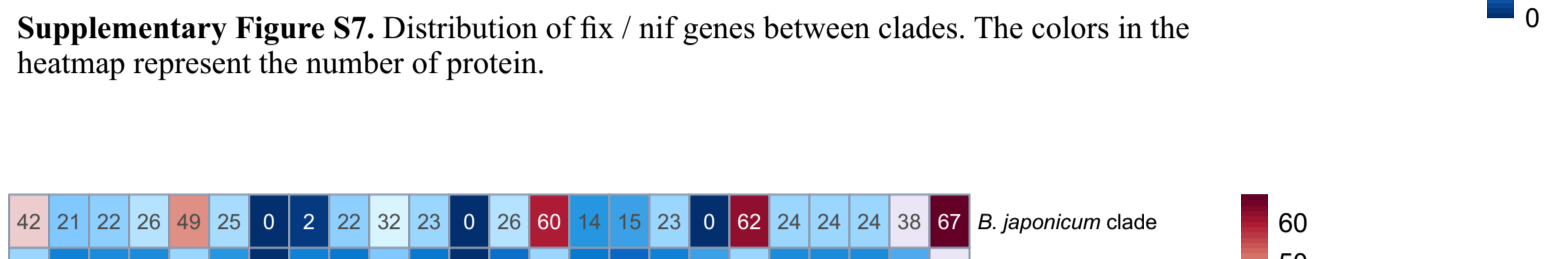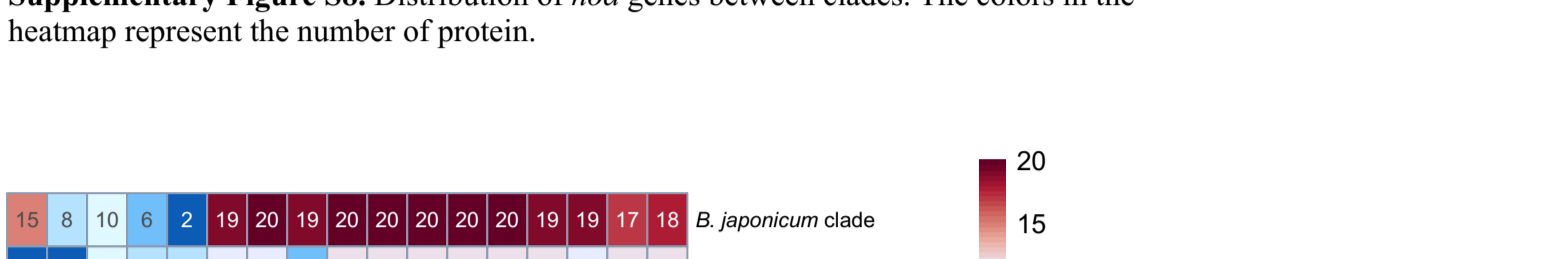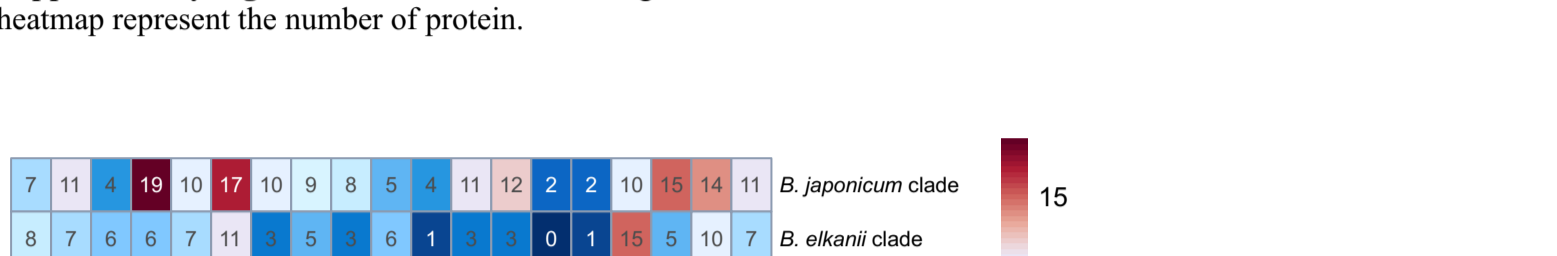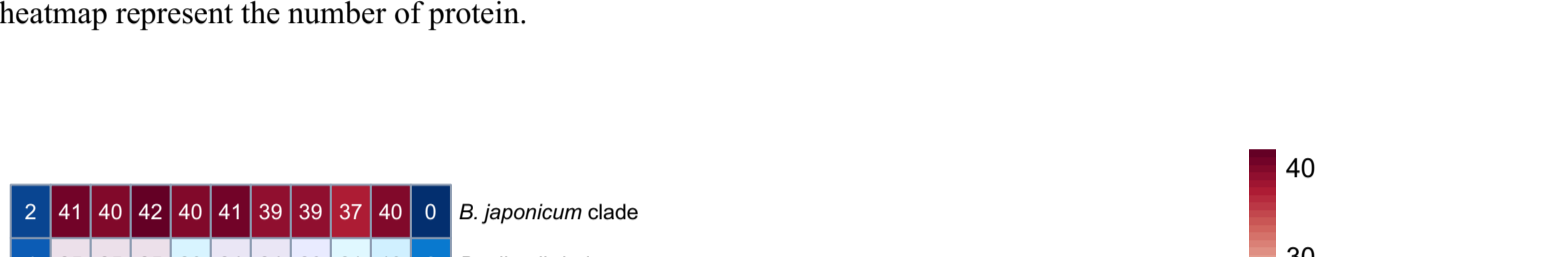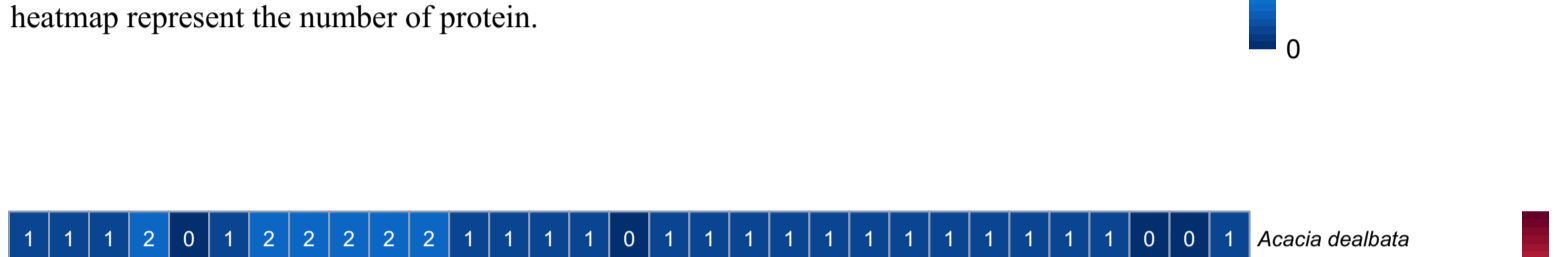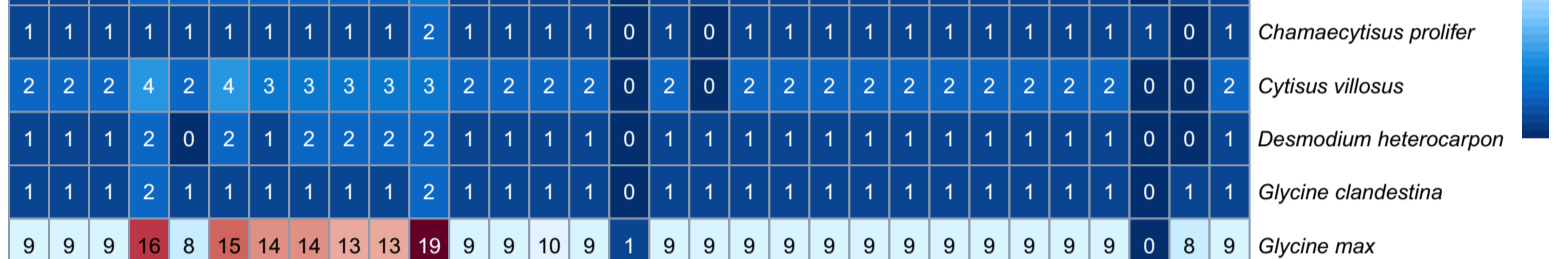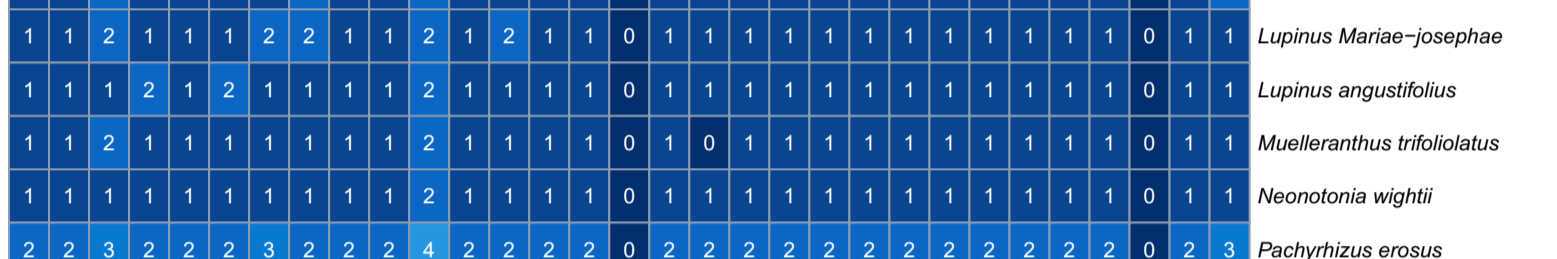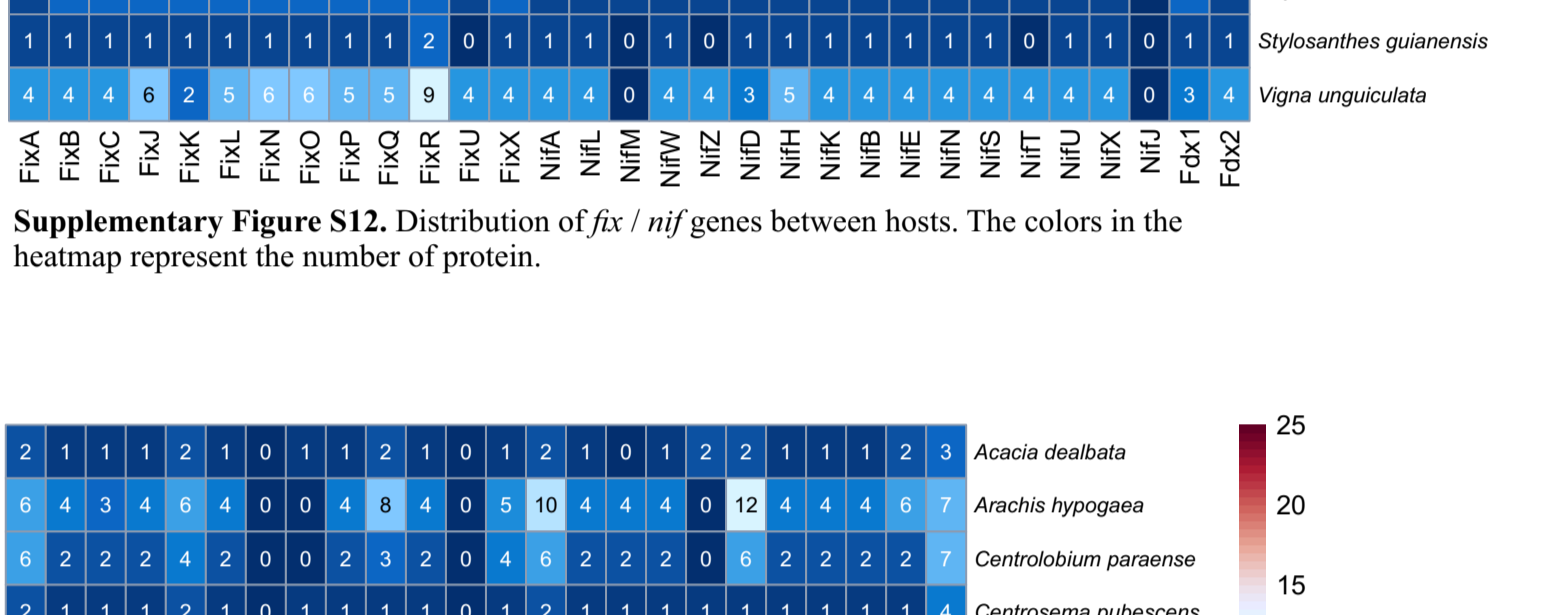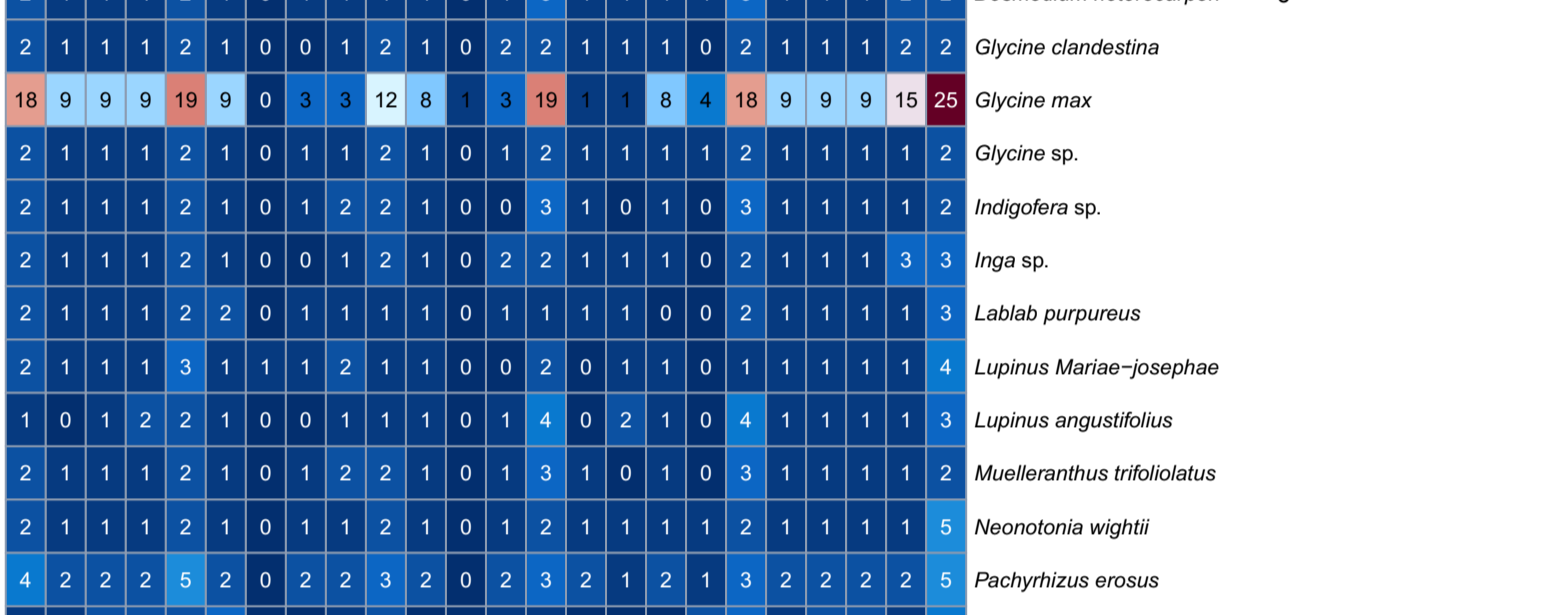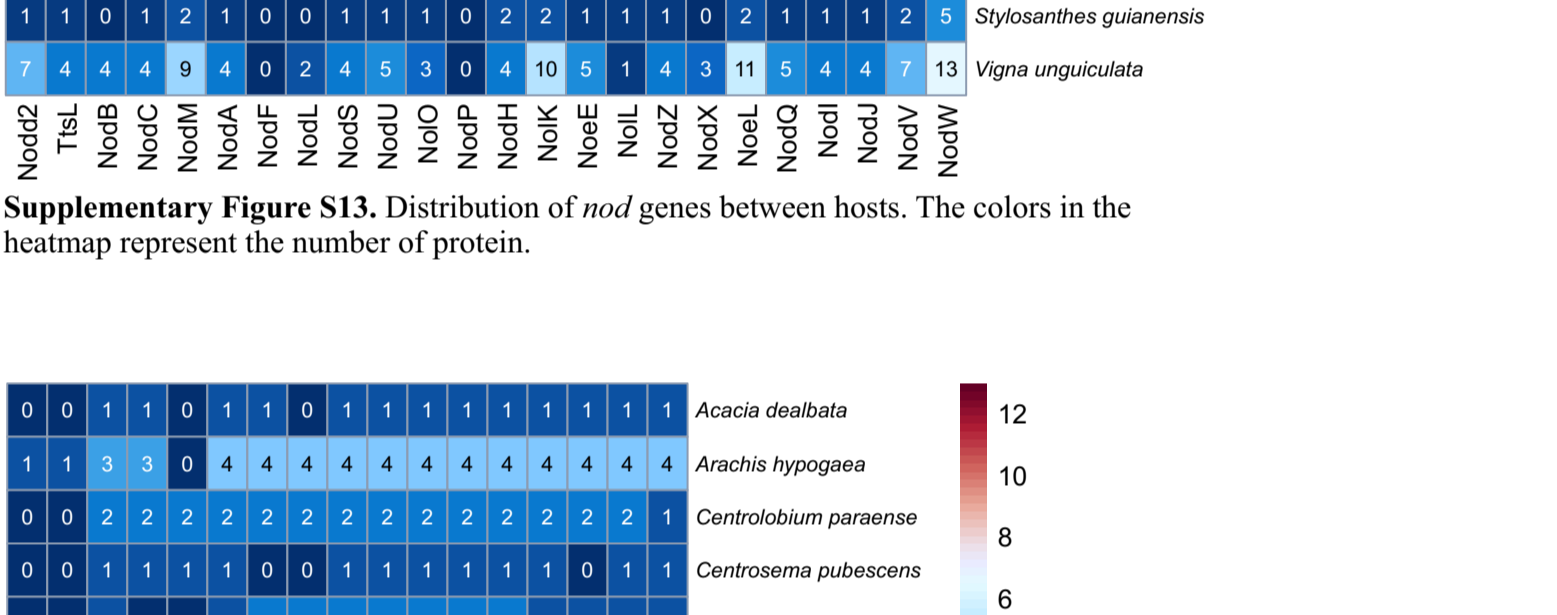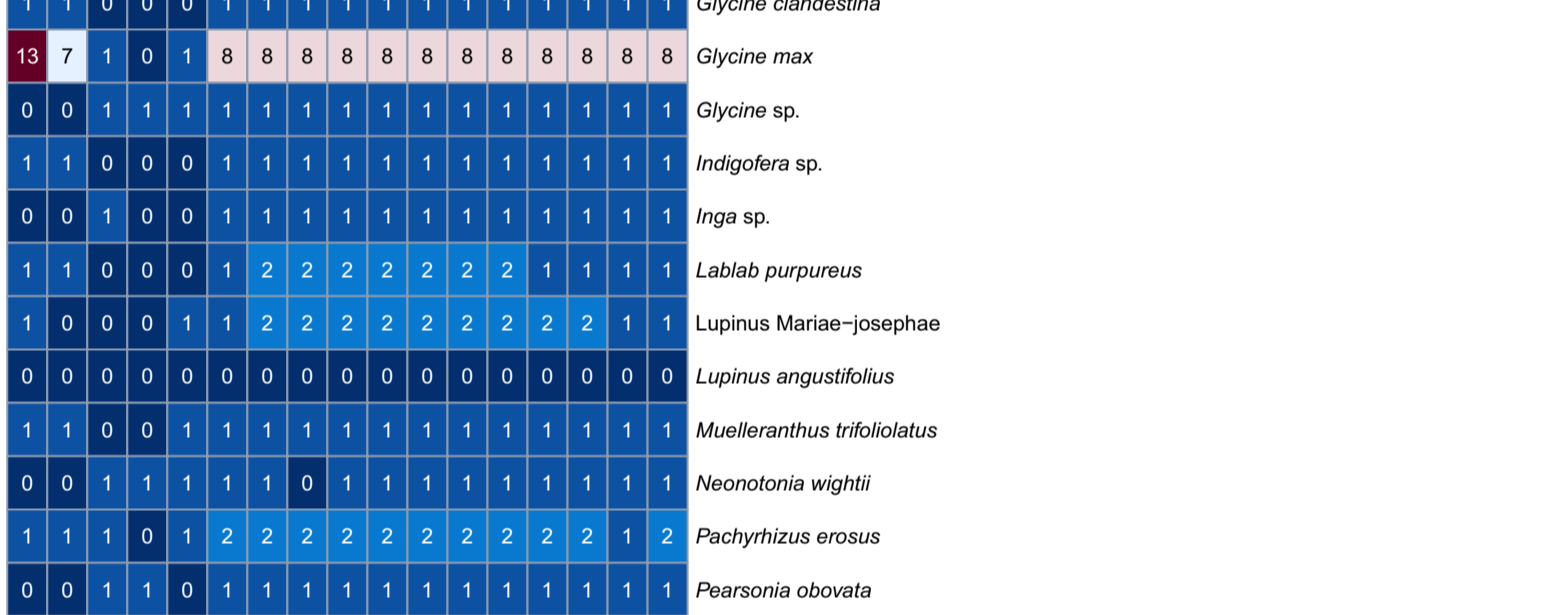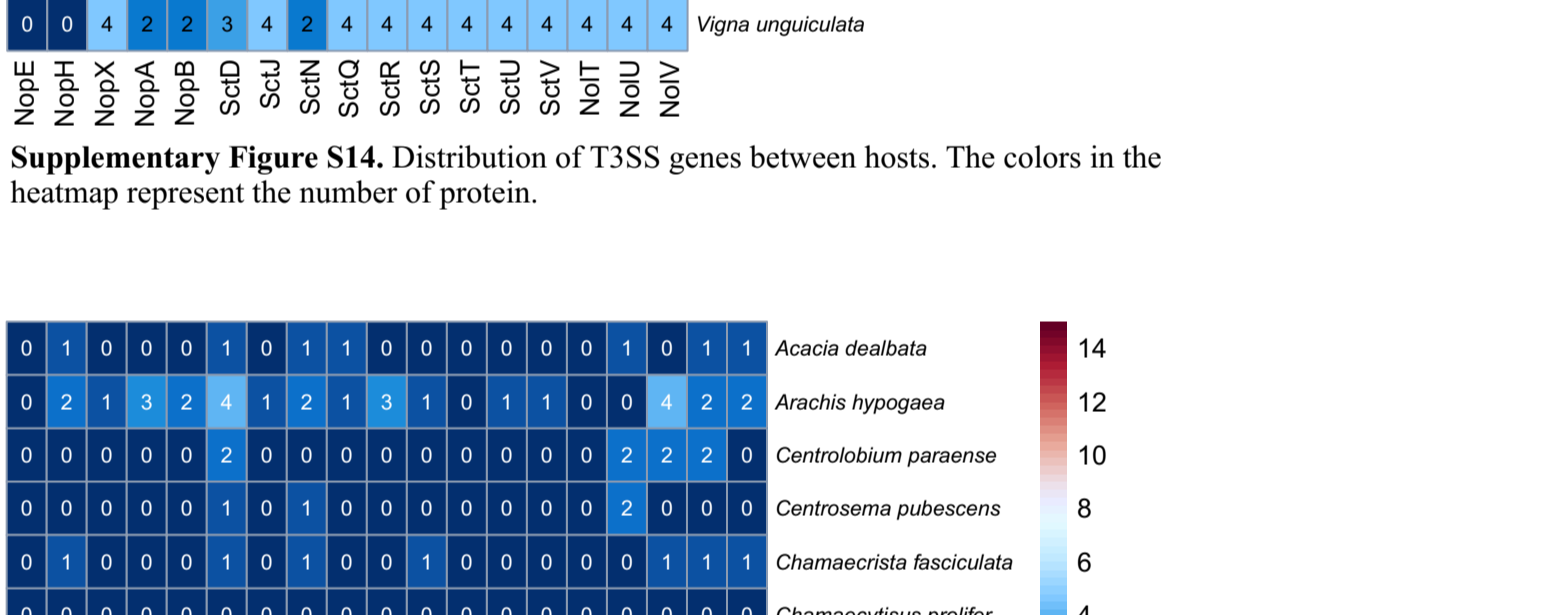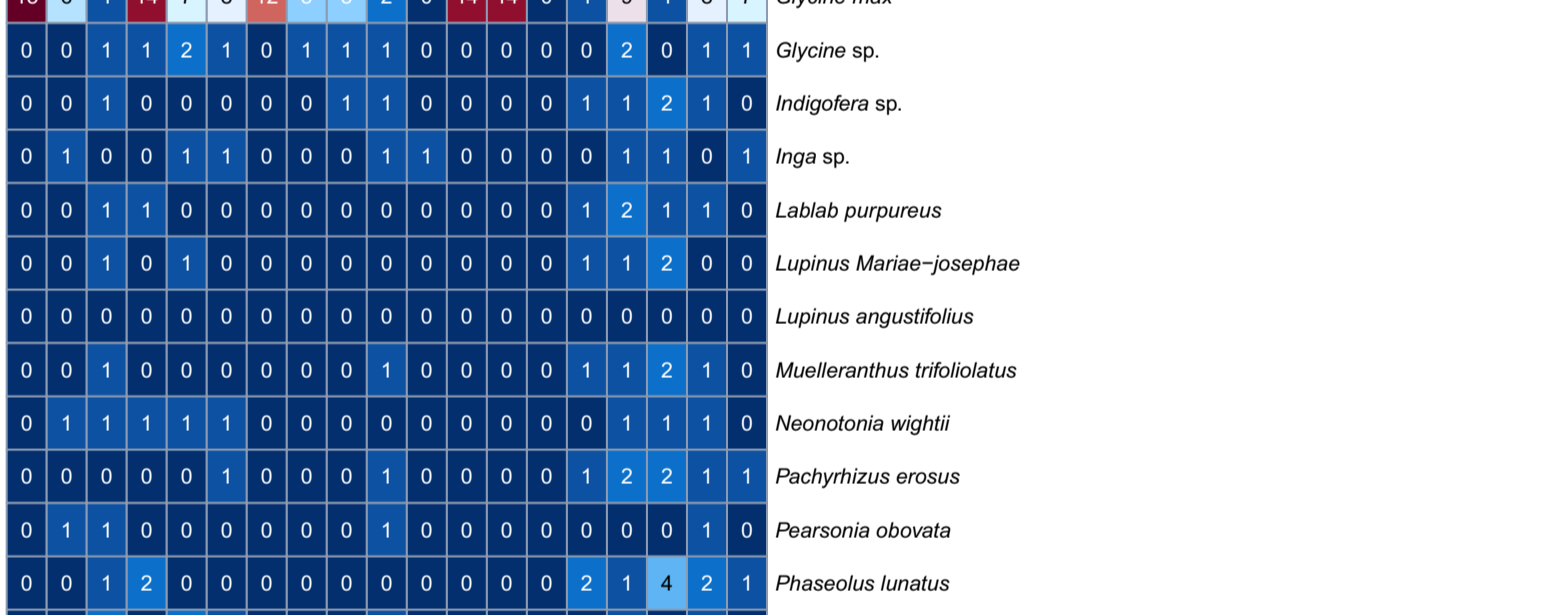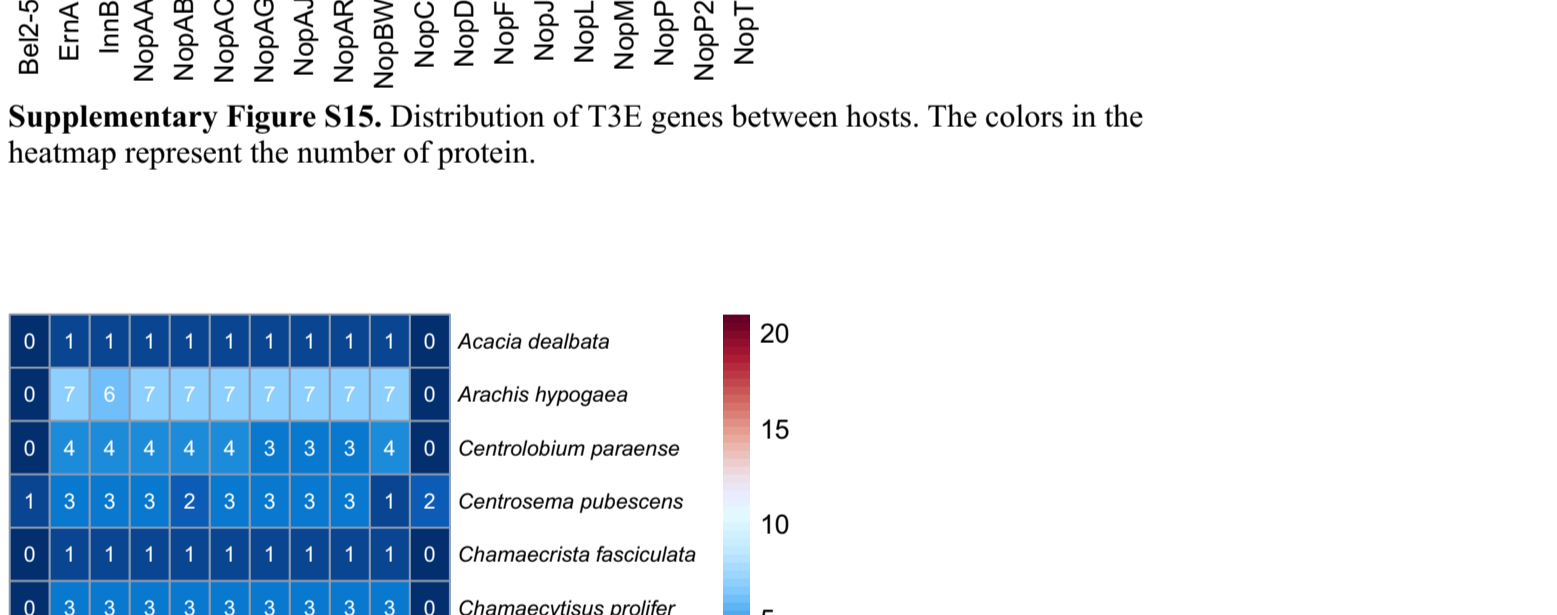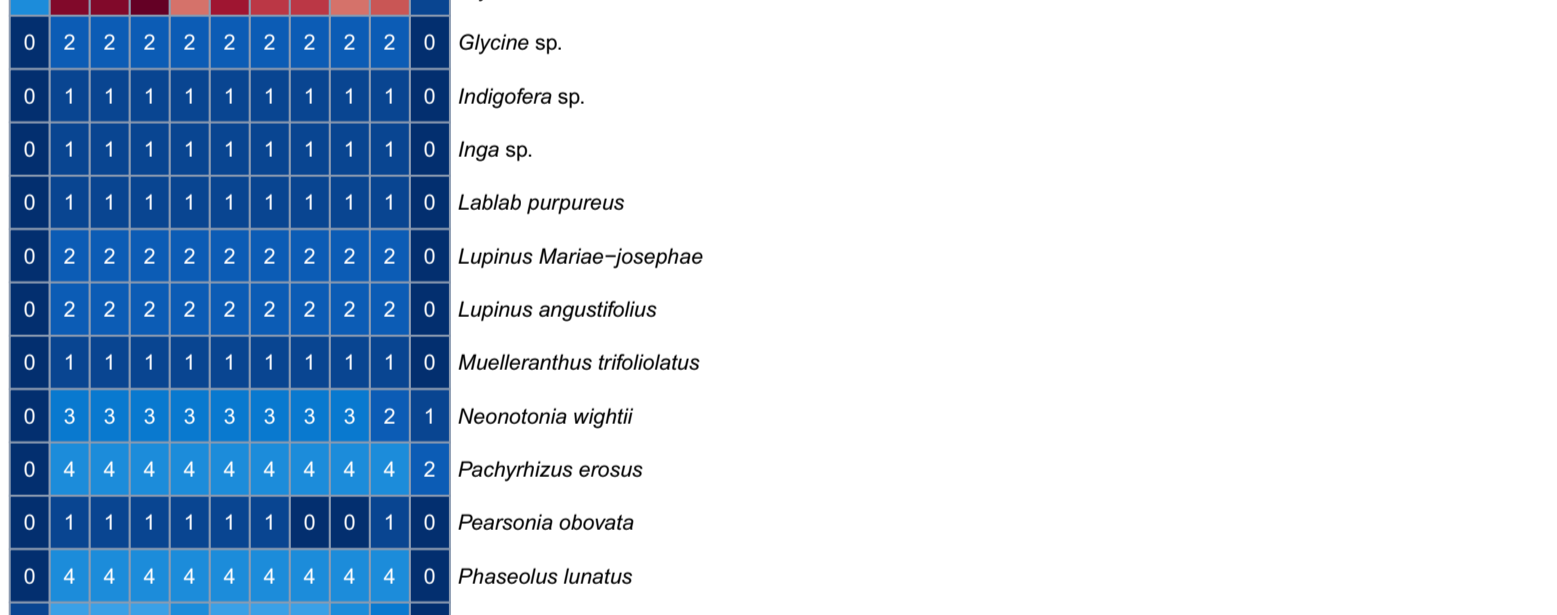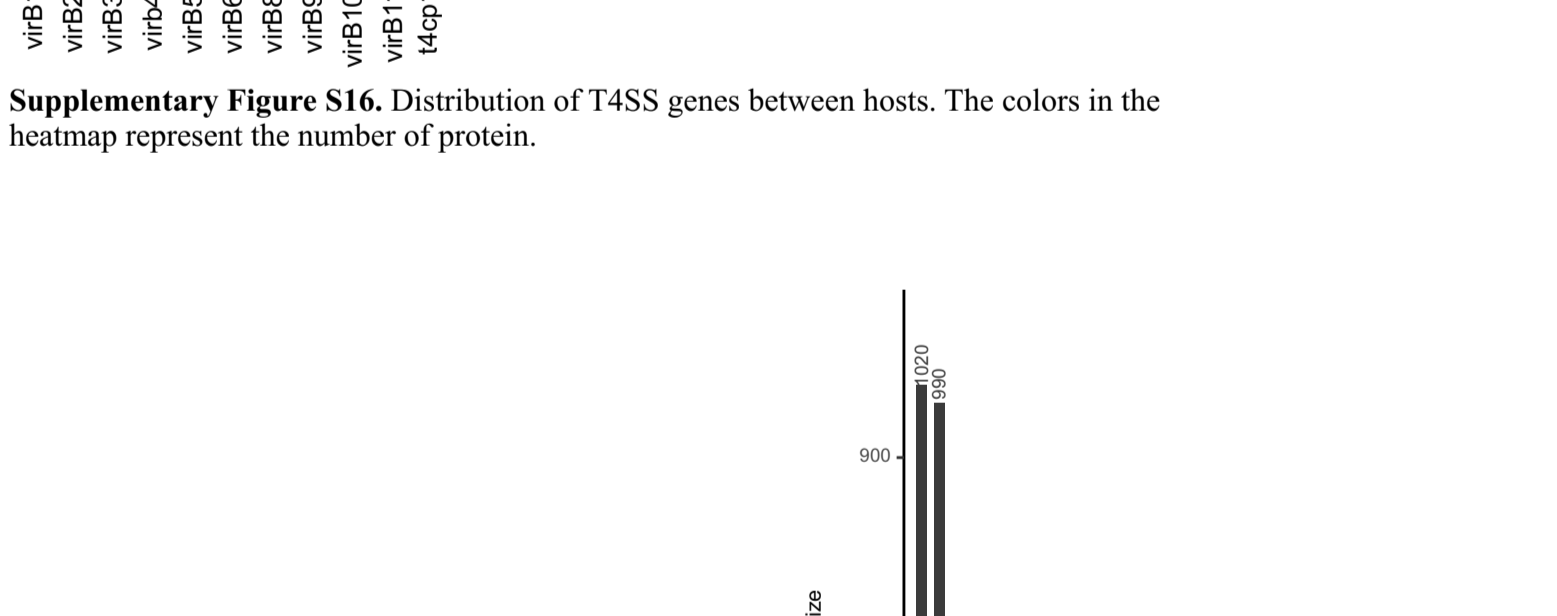

Supplement: Supplementary File 2 — (Figures S1, S2, S3, S4, S5, S6, S7, S8, S9, S10, S11, S12, S13, S14, S15, S16, S17, S18 and S19) | Heat map representing the distribution of fix/nif, nod, T3SS and their effector proteins and T4SS genes in comparison to the different hemispheres, clades (Bradyrhizboium jicamae, B. elkanii and B. japonicum) and their respective hosts. UpSet plots demonstrating the sharing of accessory genome gene clusters between species from the Northern and Southern Hemispheres. Analysis of the distribution of insertion elements in Bradyrhizobium analyzed by different geographic regions and comparisons between hemispheres. [file DataSheet2.pdf]
