# Supplementary material for: Pangenome analysis indicates evolutionary origins and genetic diversity: emphasis on the role of nodulation in symbiotic Bradyrhizobium
Source: Front Plant Sci. 2025 Apr 2;16:1539151. doi: 10.3389/fpls.2025.1539151 (PMC12000093; doi:10.3389/fpls.2025.1539151)
Supplement: Supplementary File 3 — Instruction guide for using the interactive map. [file DataSheet3.docx]

README

**Interactive Map Installation**

Article Title:

Pangenome analysis indicates evolutionary origins and genetic diversity: emphasis on the role of nodulation in symbiotic *Bradyrhizobium*

Authors:

1. Leonardo Araujo Terra

1. Milena Serenato Klepa

2. Marco Antonio Nogueira

2. Mariangela Hungria

Institutions:

1. CNPq, Ed. Telemundi II, SAUS Quadra 01 Lotes 1 e 6, CEP Brasília, Federal District, Brazil

2. Embrapa Soja, Soil Biotechnology Laboratory, C.P. 4006, 86.085-981, Londrina, Paraná, Brazil

* Corresponding author: mariangela.hungria@embrapa.br; biotecnologia.solo@hotmail.com

Program developed by Leonardo Araujo Terra ([leonardoterra@hotmail.com.br](mailto:leonardoterra@hotmail.com.br))

**Interactive Map**

Proteins highlighted in black indicate the presence of a gene in the genome, while those shown in gray indicate its absence. The numbers inside each box correspond to the number of genes present in each category.

**Availability of data and materials**

The data that support the conclusions of this study are available on the ZENODO platform (https://zenodo.org/doi/10.5281/zenodo.12789079), and are publicly available.

Instructions:

1. Extracting Files

=========================

The folder contains all the necessary files to view the interactive map of *Bradyrhizobium* markers.

- Use a decompression software (such as WinRAR, 7-Zip, or the native utility of your operating system) to extract the files.

- Make sure to extract all files into the same folder to ensure all resources (images, HTML, etc.) are correctly referenced.

2. Viewing the Map

=========================

After extracting the files, follow the instructions below to view the interactive map:

- Locate the interactive_map.html file in the folder where the files were extracted.

- Open the interactive_map.html file in a web browser (we recommend using Google Chrome).

When you open the HTML file in the browser, you will see an interactive map with markers indicating different Bradyrhizobium species. Click on the markers to view the information and images associated with each species.

Important Notes:

=========================

The program was tested only on Google Chrome and will likely work on Mozilla Firefox and Microsoft Edge.

The figures must be in the same folder as the HTML file for the interactive map program to function properly.

For more information or support, please contact the authors of the article.

Thank you for your interest in our work!
